# Incidence, risk factors and etiological agents in surgical site infections in a developing country

**DOI:** 10.1186/1753-6561-5-S6-P194

**Published:** 2011-06-29

**Authors:** E Alp, F Elmali, S Ersoy, C Kucuk, B Tucer, M Doganay

**Affiliations:** 1 Erciyes University, Kayseri, Turkey

## Introduction / objectives

There is limited data about the epidemiology of SSIs in developing countries.To investigate incidence rates of SSIs, risk factors, etiological agents and antimicrobial resistance rates of pathogens in a developing country.

## Methods

Prospective surveillance of SSIs was performed during May 2005 and April 2009 in neurosurgery (NS) and general surgery (GS) unit. All patients who had gallbladder (CHOL), colon (COLO), gastric (GAST), small bowel (SB) and bile duct, liver or pancreatic surgery (BILI) in GS and craniotomy (CRAN), ventricular shunt (VSHN) and spinal fusion (FUSN) surgery in NS were included.

## Results

SSI was determined in 415 (10.8%) patients in GS and 146 (4%) of patients in NS.SSI rates were 4%,16.8%,6%,16.4% and 14% in CHOL,COLO,GAST,SB and BILI, respectively in GS.In NS, SSI rates were 4%, 4.8% and 4.5% in CRAN,VSHN and FUSN.Cefazolin was used in 780 (49%) operations in GS, 1266 (95%) of operations in NS for antimicrobial prophylaxis.Broad spectrum antibiotics were administered in the rest of the patients.Antimicrobial prophylaxis (AMP) were administered for >24 h in 69% and 64% of patients in GS and NS, respectively.The most significant risk factors for SSIs were total parenteral nutrition, transfusion and presence of drain in GS, and total parenteral nutrition, transfusion, stress ulcer prophylaxis, presence of drain and foreign material in NS.The most common pathogen was *Escherichia coli* in GS and *Acinetobacter baumannii* in NS.Isolated pathogens were multiresistant, with 58% quinolone resistance in *E.coli* and 67% imipenem resistance in *A.baumannii*.

## Conclusion

Surveillance of surgical site infections is one of the most important infection control issue. Prolonged use of AMP and use of broad spectrum antibiotics are associated with emergence of resistant bacterial strains.

## Disclosure of interest

None declared.

